# The role of methylenetetrahydrofolate reductase C677T gene polymorphism as a risk factor for coronary artery disease: a cross-sectional study in the Sidoarjo Regional General Hospital

**DOI:** 10.11604/pamj.2022.41.212.24916

**Published:** 2022-03-15

**Authors:** Hairudi Sugijo, Djanggan Sargowo, Edi Widjajanto, Rochmad Romdoni

**Affiliations:** 1 Cardiology and Vascular Medicine Department, Sidoarjo Regional General Hospital, Sidoarjo, Indonesia,; 2 Internal Medicine Department, Faculty of Medicine, Brawijaya University, Malang, Indonesia,; 3 Clinical Pathology Department, Faculty of Medicine, Brawijaya University, Malang, Indonesia,; 4 Cardiology and Vascular Medicine Department, Faculty of Medicine, Airlangga University, Surabaya, Indonesia

**Keywords:** Coronary artery disease, hyperhomocysteinemia, homocysteine, gene polymorphism, risk factor

## Abstract

**Introduction:**

hyperhomocysteinemia (HHcy) may contribute to an increased risk of coronary artery disease (CAD). The underlying mechanisms are not well understood, but other than dietary intake factors, hyperhomocysteinemia may genetically result from a methylenetetrahydrofolate reductase (MTHFR) C677T gene polymorphism. A cross-sectional study was performed to assess whether this mutation was a potential genetic risk factor for CAD.

**Methods:**

this cross-sectional study was performed on 30 CAD patients and 30 normal healthy controls at Sidoarjo Regional General Hospital. The polymorphisms of the MTHFR C677T gene was assessed by polymerase chain reaction (PCR), and plasma homocysteine was measured by chemiluminescence immunoassay (CLIA) and then compared between CAD patients and control subjects by the multivariate logistical regression model.

**Results:**

results from an independent sample t-test analysis showed that plasma homocysteine concentrations were significantly higher in CAD patients compared to the control group individuals (13.91 ± 4.55 μmol/L vs 10.97 ± 3.45 μmol/L; p<0.05). There were no significant correlations between MTHFR C677T gene polymorphism and other risk factors, such as age at diagnosis with acute coronary syndrome, sex, smoking, lipid profile, diabetes, hypertension, C-reactive protein (CRP), creatinine, and homocysteine (p>0.05). In multivariate analysis models, the C677T genotype frequencies were insignificantly different between CAD patients and control subjects (p>0.05). Meanwhile, the results of adjusted odds ratio (aOR), 95% confidence interval (CI), and p-value for homocysteine, age, and smoking were aOR: 1.264, 95% CI : 1.042-1.535, p = 0.018; aOR: 0.916, 95% CI: 0.842-0.997, p = 0.043, and aOR: 5.428, 95% CI 1.532-19.226, p = 0.009, respectively. Homocysteine, age, and smoking were significantly different between CAD patients and control subjects (p<0.05).

**Conclusion:**

hyperhomocysteinemiais significantly correlated with an increased risk of CAD, but MTHFR C677T gene polymorphism might not contribute to increased CAD risk.

## Introduction

Both clinical and experimental studies have suggested that an elevated plasma homocysteine level is a significant risk factor for coronary artery disease (CAD) [1-4]. The proposed mechanism by which hyperhomocysteinemia (HHcy) causes atherothrombosis is by impairing endothelial function, increasing oxidative stress, inducing inflammation, increasing *endoplasmic reticulum* (ER) *stress* and apoptosis, increasing autoimmune reactions, and increasing the coagulation cascade [5-10]. Homocysteine levels are affected by age, gender, folate, vitamin B, and renal and liver function [11]. Other than nutritional deficiencies, plasma homocysteine concentrations are determined by genetic factors. The methylenetetrahydrofolate reductase (MTHFR) C677T gene polymorphism is the most commonly studied form of genetic hyperhomocysteinemia, in which the enzyme MTHFR becomes thermolabile and functionally impaired [12-14]. Homozygous TT of MTHFR C677T gene polymorphism is associated with HHcy, especially in the presence of low plasma folate levels [15,16].

The results of the meta-analysis showed strong evidence of a correlation between MTHFR C677T gene polymorphism with hyperhomocysteinemia and CAD in Asia and the Middle East, but not in North America, Europe, and Australia [17]. However, the result of this meta-analysis raises the question of whether there are confounding effects of lifestyle, demographic and/or ethnicity influence on the correlation between the MTHFR C677T gene polymorphism with hyperhomocysteinemia and CAD. Sidoarjo Region is located in the north east part of Java where the local diet is dominated by grains rich in vitamins B and folate. As vitamins B and folate are cofactors in Hcy metabolism that can significantly reduce Hcy concentration, it was hypothesized that the incidence of hyperhomocysteinemia in Sidoarjo was related to polymorphisms of MTHFR C677T. This known genetic predisposition may contribute to better health care strategic planning such as providing folate supplementation in high-risk patients. Therefore, we performed a cross-sectional study to investigate the correlation between MTHFR C677T gene polymorphism and plasma homocysteine levels in the presence of CAD in patients from Sidoarjo Region.

The aim of the present study was to identify whether a high incidence of hyperhomocysteinemia in CAD patients could be determined by individual polymorphisms of MTHFR C677T, as the diet in Sidoarjo are beneficial to the reduction of Hcy levels. In addition, multiple factors that contribute to CAD were assessed and used to evaluate its correlation with MTHFR C677T gene polymorphism.

## Methods

**Study design:** to investigate the distribution characteristics of MTHFR gene polymorphism, a cross-sectional study was conducted. Following the sample size calculation, subjects who met the following inclusion and exclusion criteria were recruited by purposive sampling from individuals who visited the outpatient clinic of the Sidoarjo Regional General Hospital in East Java, Indonesia for a period of 6 months between July 2017 and December 2017. Blood samples taken were stored until plasma Hcy and C677T MTHFR gene polymorphism investigations were performed on the blood samples as per protocol. Statistical analyses were then performed using Statistical Package for the Social Sciences (SPSS) version 17.0 statistics software.

**Sample size calculation:** the sample size formula for unpaired numerical analytical research was used to calculate the sample size. According to the admissible error of 5% and confidence of 1-α=0.95, the result showed that the sample size was 27 at minimal for each group.

**Study population:** in the present retrospective study, 30 CAD cases and 30 controls were recruited by purposive sampling from individuals who visited the outpatient clinic of the Sidoarjo Regional General Hospital in East Java, Indonesia for a period of 6 months between July 2017 and December 2017. Participants who met the following inclusion criteria were enrolled: CAD cases were defined as patients with a history of documented ST-elevation acute coronary syndrome (STE-ACS). Controls were healthy individuals who had never suffered from acute coronary syndrome with normal electrocardiogram, negative treadmill test results for myocardial ischemia, and without any major illnesses. The exclusion criteria were as follows: subjects who suffered from cancer, liver disease, renal insufficiency, blood disease, and thyroid dysfunction, pregnant women, and those consuming folic acid and vitamin B_12_ supplements were excluded. The participating subjects were given information about the research procedures, and their written informed consent was obtained.

**Data collection:** the examination included the measurement of systolic blood pressure (SBP) and diastolic blood pressure (DBP). Bodyweight and height were measured with standard techniques. Hypertension was defined as the current use of antihypertensive medications, SBP >140 mmHg, or DBP >90 mmHg. Body mass index (BMI) was calculated as weight (kg) divided by the square of height (m). Smoking was defined as smoking in the last 5 years. Fasted blood samples were taken in the morning after 12 h of fasting in the lying down position and collected in a lithium heparinized BD Vacutainer tubes. Samples were centrifuged at 2000 rpm for 10 min, and 0.5 ml aliquots were added and stored at -20°C until they were assayed with a Cobas c-501 analyzer (Roche Diagnostics) for the determination of Hcy, folate, and vitamin B_12_. Another fresh plasma sample was used for the determination of fasting glucose, 2 h postprandial glucose, C-reactive protein (CRP), total cholesterol, triglyceride (TG), high-density lipoprotein (HDL), and low-density lipoprotein (LDL) with a Cobas e-601 analyzer (Roche Diagnostics). Hyperhomocysteinemia was defined as Hcy levels greater than 15 μmol/L, while low Hcy was defined as Hcy levels less than 5 μmol/L (11). All data from selected samples were used. Bias in data might occurred as the control group was not undergone coronary angiography as the gold standard to exclude the CAD diagnosis. There were no missing data in participants as purposive sampling was used. Furthermore, loss to follow-up cases was replaced by newly recruited subjects.

**DNA (deoxyribonucleic acid) extraction and molecular analysis:** an examination of single-nucleotide polymorphisms (SNP) of the MTHFR C677T gene (cat. no rs1801133) was carried out using venous blood from the sample and control groups, taken using BD Vacutainer® spray-coated K2EDTA tubes and stored at -80°C. DNA was isolated from 200 µL of venous blood using a High Pure PCR Template Preparation kit obtained from Roche (cat. no 11796828001). From the DNA isolation result, genotyping was examined using real-time polymerase chain reaction (real-time polymerase chain reaction (PCR) using LightCycler® 480, Roche Applied Science, Mannheim, Germany). Primers and probes are as follows: primary sequence, 5'-TGG CAG GTT ACC CCA AAG G-3 '(forward) and 5'-TGA TGC CCA TGT CGG TGC-3' (reverse), and sequence hybridization probe, 5'- TGA GGC TGA CCT GAA GCA CTT GAA GCA CTT GAA PRICE GAA GGT GTC T-3'-Flu and 5'-LC-640-CGG GAG CCG ATT TCA TCA T-3'-PHO (TIB Molbiol-Roche, Berlin Germany; cat. no 03003248001). The real-time PCR examination procedure begins with making a program protocol consisting of four stages, namely, denaturation of DNA samples and enzyme activation, PCR amplification of target DNA, analysis by melting curve at 95°C, 40°C, and 85°C, and cooling of the instrument. This was followed by preparing parameter-specific reagents, DNA control solution, and LightCycler® reaction mix. The program is then run, and the results are analyzed according to the instructions in the LightMix Kit MTHFR C677T (TIB Molbiol, Berlin Germany; cat.no 40-0095-16).

**Statistical analysis:** data were statistically analyzed using Statistical Package for the Social Sciences (SPSS) version 17.0 (SPSS 17.0; SPSS Inc., Chicago, IL, USA). Numerical variables were expressed as the mean ± standard deviation (SD), while categorical variables were expressed as a percentage. Testing for normal data distributions was performed using the Shapiro-Wilk test for numerical data and using the Chi-square test for categorical data. Intergroup comparisons were made using an independent Student´s t-test for parametric data, the Mann-Whitney U test for continuous non-normal distributions data, and Chi-square test for nominal data. Correlations between variables were evaluated with the Spearman correlation test. The Hardy-Weinberg equilibrium test evaluated the consistency of the genotype frequency of the MTHFR C677T population (CC, CT, TT). The odds ratio of CAD occurrence in the homozygous genotype group was evaluated using a Chi-square test with a 95% confidence interval. Differences were considered significant if the P-value was less than 0.05 (p<0.05). Logistic regression for univariable and multivariable analysis was used to analyze the correlation between risk factors and CAD.

**Ethical consideration:** the Health Research Ethics Committee of the Wijayakusuma Surabaya University approved the study (ethical clearance letter no. 10198/SLE/FK/UWKS). Each participant signed an informed consent form before completing the examinations.

## Results

There was a total of 111 patients with a history of documented ST-elevation acute coronary syndrome (STE-ACS) and 75 normal patients in the cardiology outpatient clinic of the Sidoarjo Regional General Hospital in East Java, Indonesia for a period of 6 months between July 2017 and December 2017. We reviewed the medical record to select patients that met the inclusion and exclusion criteria until the targeted number was reached by consecutive sampling (30 patients and 30 controls). The selected patients were included 30 CAD patients (26 males and 4 females) aged 21-59 (44.6 ± 8.935) years and 30 controls (23 males and 7 females) aged 33-75 (48.83 ± 8.76) years. No loss of follow-up because the data was collected and recorded directly at one time once the patients have given consent.

**Patients characteristics:** the differences in the mean age and the number of males and females in the two groups were not statistically significant. The CAD group had a significantly higher homocysteine level (13.91 ± 4.55 mmol/L vs 10.97 ± 3.45 mmol/L; p<0.05) and had a significantly higher percentage of smoking or stopping smoking ≤5 years compared to the control group (73.3% vs 33.3%; p <0.05) (Table 1), indicating homocysteine and smoking as significant risk factors among CAD patients. There were insignificantly higher prevalence of CC and TT genotypes in the cases and higher CT genotypes in the controls, indicating that MTHFR C677T gene polymorphism did not contribute to increased CAD risk (Table 1).

**Table 1:** clinical characteristics of coronary artery disease (CAD) and control study groups

Characteristics	CAD	Control	P-value
Male	26 (86.7%)	23 (76.7%)	0.505
Female	4 (13.3%)	7 (23.3%)	
Age^c^	44.6 ± 8.935	48.83 ± 8.76	0.069
BMI	24.80 ± 3.56	24.61 ± 4.66	0.862
Smoking	^a^22 (73.3%)	^a^10 (33.3%)	0.004, OR=5,50 (95% CI 1.81-16.68)
	^b^8 (26.7%)	^b^20 (66.7%)	
Hypertension	9 (30.0%)	4 (13.3%)	0.210
Cholesterol (mg/dl)	180.17 ± 37	189.17 ± 40.75	0.374
TG (mg/dl)	148.60 ± 57.35	144.17 ± 71.46	0.515
LDL (mg/dl)	120.97 ± 30.71	129.20 ± 36.99	0.464
HDL (mg/dl)	41.50 ± 11.74	44.2 ± 12.8	0.529
Fasting glucose (mg/dl)	98.70 ± 13.41	99.77 ± 8.89	0.340
2h PP glucose (mg/dl)	129.10 ± 21.02	129.97 ± 22.11	0.888
CRP (mg/dl)	2.31 ± 7.30	0.34 ± 0.46	0.174
Creatinine (mg/dl)	0.94 ± 0.17	0.96 ± 0.21	0.664
Homocysteine (μmol/L)	13.91 ± 4.55	10.97 ± 3.45	0.004
Folate (ng/ml)	9.81 ± 3.51	11.34 ± 3.53	0.097
Vitamin B12 (ng/L)	416.88 ± 322.82	417.01 ± 190.37	0.641
MTHFR C677T gene polymorphism			
CC	25 (83.3%)	22 (73.3%)	0.330
CT	4 (13.3%)	8 (26.7%)	
TT	1 (3.3%)	0	

Number in mean ± SD, n (%); ^a^: smoking or stop smoking ≤5 years; ^b^: nonsmoking/stop smoking >5 years; ^c^: age when diagnosed with acute coronary syndrome; BMI: body mass index; TG: triglyceride; LDL: low-density lipoprotein; HDL: high-density lipoprotein; PP: postprandial; CRP: C-reactive protein

**Correlations between MTHFR C677T gene polymorphism and other risk factors:** Table 2 summarizes the correlations between MTHFR C677T gene polymorphism and age of diagnosis with an acute coronary syndrome, sex, smoking, lipid profile, diabetes, hypertension, CRP, creatinine, and homocysteine. No significant correlations were found between MTHFR C677T gene polymorphism and other risk factors, indicating that MTHFR C677T gene polymorphism did not correlate with the development of CAD risk factors.

**Table 2:** correlation of MTHFR C677T gene polymorphism with other risk factors

Characteristics	r	P value
Sex	0.02	0.87
Age	0.02	0.89
Body mass index (BMI)	0.03	0.84
Smoking	-0.13	0.33
Hypertension	0.22	0.09
Total cholesterol	-0.04	0.74
Triglyceride (TG)	-0.08	0.525
Low-density lipoprotein (LDL)	-0.05	0.68
High-density lipoprotein (HDL)	-0.07	0.58
Fasting glucose	-0.09	0.49
2 h postprandial glucose	-0.14	0.27
C-reactive protein (CRP)	-0.09	0.51
Creatinine	0.06	0.63
Homocysteine	0.08	0.55
CAD/control	-0.11	0.4
CAD/control^a^	-0.16	0.22

a : partial correlation with the adjustment: smoking, homocysteine; CAD: coronary artery disease

**Distribution of MTHFR C677T genotypes:** Table 3 shows the distribution of MTHFR C677T genotypes and the allele frequencies of the study groups, and no significant differences were found between cases and controls. The rates were compatible with the Hardy-Weinberg equilibrium. There were higher prevalence of the CC and TT genotypes among the cases but higher CT genotypes and T alleles among the controls, indicating there was no significant correlation between MTHFR C677T gene polymorphism with CAD.

**Table 3:** MTHFR C677T genotype in coronary artery disease (CAD) patients and control individuals

MTHFR C677T SNP	CAD		Control	
	n	(%)	n	(%)
CC	25	(83.3%)	22	(73.3%)
CT	4	(13.3%)	8	(26.7%)
TT	1	(3.3%)	0	(0%)
HWE^a^	2.02		0.7	
OR^b^		2.65 (0.1-68.3)		
P value		0.56		
	Allele			
Allele C	54 (90%)		52 (86.67%)	
Allele T	6 (10%)		8 (13.3%)	

a the (HWF) Hardy-Weinberg equilibrium, consistent if p > 0.05; ^b^OR = odd ratio for genotypes comparing homozygotes

**Relationships between risk factors and CAD:** in the logistic regression analysis of the relation of risk factors and CAD, homocysteine had a significant correlation with CAD (OR 1.26; 95% CI 1.042 - 1.535; p<0.05). Patients who were still smoking and stopped smoking less than 5 years prior had a higher incidence of CAD (OR 5.428; 95% CI 1.532-19.226; p<0.05). Together, this indicates that homocysteine and smoking might be strong independent risk factors of CAD (Table 4).

**Table 4:** relationships between risk factors and coronary artery disease (CAD)

Univariable and multivariable predictors of CAD				
Predictors	Univariable analysis		Multivariable analysis	
	OR (95% CI)	p value	OR (95% CI)	p Value
Gender (male)	1.978 (0.513-7.635)	0.322		
Age	0.944 (0.866-1.006)	0.078	0.916 (0.842-0.997)	0.043
BMI	1.009 (0.891-1.142)	0.889		
Smoking	5.500 (1.813-16.681)	0.003	5.428 (1.532-19.226)	0.009
Hypertension	0.643 (0.221-1.873)	0.418		
Cholesterol	0.994 (0.980-1.007)	0.370		
TG	1.001 (0.993-1.009)	0.788		
LDL	0.993 (0.977-1.008)	0.348		
HDL	0.981 (0.940-1.025)	0.396		
Fasting glucose	0.992 (0.948-1.037)	0.713		
2h pp glucose	0.998 (0.975-1.022)	0.874		
CRP	1.738 (0.633-4.774)	0.284		
Creatinine	0.464 (0.030-7.103)	0.581		
Homocysteine	1.253 (1.054-1.488)	0.010	1.264 (1.042-1.535)	0.018
Folate	0.880 (0.755-1.026)	0.102	0.973 (0.812-1.167)	0.772
Vitamin B_12_	1.000 (0.998-1.002)	0.998		
MTHFR C667T gene (CT or TT)	0.550 (0.157-1.931)	0.351		

TG: triglyceride; LDL: low-density lipoprotein; HDL: high-density lipoprotein; PP: postprandial

## Discussion

Hyperhomocysteinemia (HHcy) has been identified as a novel risk factor for coronary artery disease (CAD). This research was conducted to assess whether a methylenetetrahydrofolate reductase (MTHFR) C677T gene polymorphism resulted in hyperhomocysteinemia as a potential genetic risk factor for CAD. The findings in our present study are consistent with previous studies, which showed that HHcy is related to CAD as a strong independent risk factor [1-4].

Each increase in homocysteine level of 1 μmol/L will increase the risk of CAD by 1.2 times (OR 1.26; 95% CI 1.042 - 1.535 (p<0.05) (Table 4). Homocysteine is metabolized into a thioester form, namely, homocysteine thiolactone (Hcy-thiolacton), which is more toxic and can cause the formation of N-homocysteinylation of LDL (N-Hcy-LDL) and N-homocysteinylation of fibrinogen (N-Hcy-fibrinogen) [18-20]. N-homocysteinylation of LDL (N-Hcy-LDL) increases the risk of CAD development through an increased reactive oxygen species (ROS) formation [5,8,21,22], inflammatory reactions [23-25], ER stress and cell apoptosis [9,10,26,27] autoimmune reactions [7,28], and the coagulation cascade [6,29]. N-homocysteinylation of fibrinogen makes fibrin more difficult to be lysed, thereby increasing the risk of thrombosis [28,30]. In this study, the group that smoked or stopped smoking ≤5 years had a risk of experiencing CAD 5.5 times greater than the group who did not smoke or stopped smoking >5 years (Table 1). The results of this study are in accordance with a report from the World Health Organization, showing that smoking is responsible for 10% of cardiovascular disease events [31]. Cigarette smoking increases ROS production via NADPH oxidase, the oxidation of tetrahydrobiopterin (H4B), and the products of lipid peroxidation in endothelial cells [32,33]. Hyperhomocysteinemia is an independent risk factor for CAD, but smoking has been shown to have an amplifying effect on hyperhomocysteinemia in increasing the risk of CAD [34,35].

Some studies have found that MTHFR C677T gene polymorphism is a potential risk factor for cardiovascular disease [16,36,37]. However, in this study, we find that the prevalence of heterozygous or homozygous MTHFR genotypes is not significantly different between the cases and controls (Table 3). From the result of logistic regression analysis (Table 4), no significant correlation was found between the SNP C677T MTHFR gene and CAD (p>0.05). These results are in accordance with the result of a meta-analysis of Li *et al*. [38] and the result of several previous studies [3,39,40]. CAD is a multifactorial and polygenic disease that results from the interaction between the genetic and nutritional status of an individual; thus, the MTHFR C677T gene polymorphism does not have a direct causal role in the pathogenesis of CAD. The MTHFR C677T gene polymorphism impairs homocysteine metabolism only in the presence of inadequate folate status, as folate protects the MTHFR enzyme from becoming thermolabile by protecting it against the loss of its essential flavin cofactor [16,39,41], while folate and vitamin B12 levels in both groups in this study were still within normal limits (9.81 ± 3.51 vs 11.34 ± 3.53 ng/mL and 416.88 ± 322.82 vs 417.01 ± 190.37 ng/L; p>0.05) (Table 1). The slight prevalence of the homozygous TT of the MTHFR C677T gene polymorphism in this study was only 3.3% in the CAD group and 0% in the non-CHD group (Table 3), which could influence the absence of a causal correlation between MTHFR C677T gene polymorphism and CAD. The limitation of this study was the small size of the study population, participants not undergone coronary angiography as the gold standard of CAD diagnosis, and nutrition status was not assessed.

## Conclusion

Hyperhomocysteinemia is significantly associated with an increased risk of CAD, but MTHFR C677T gene polymorphism might not contribute to increased CAD risk in the study population at the Sidoarjo Regional General Hospital.

### What is known about this topic

- Hyperhomocysteinemia (HHcy) may contribute to an increased risk of coronary artery disease (CAD);
- Meta-analysis data showed strong evidence of a correlation between MTHFR C677T gene polymorphism with hyperhomocysteinemia and CAD in Asia and the Middle East;
- The underlying mechanisms are not well understood, but hyperhomocysteinemia may genetically result from a methylenetetrahydrofolate reductase (MTHFR) C677T gene polymorphism.

### What this study adds

- Hyperhomocysteinemiais significantly correlated with an increased risk of CAD;
- MTHFR C677T gene polymorphism might not contribute to increased CAD risk;
- Homocysteine, age, and smoking were significantly different between CAD patients and control subjects (p<0.05).
